# PREDICTORS OF WHETHER SELF-NEGLECT IS VALIDATED BY ADULT PROTECTIVE SERVICES CASE MANAGERS

**DOI:** 10.1093/geroni/igad104.0610

**Published:** 2023-12-21

**Authors:** Samantha Tuft, Farida Ejaz, Courtney Reynolds

**Affiliations:** Benjamin Rose Institute on Aging, Cleveland, Ohio, United States; Benjamin Rose Institute on Aging, Cleveland, Ohio, United States; Benjamin Rose Institute on Aging, Cleveland, Ohio, United States

## Abstract

The most reported allegation of abuse to Adult Protective Service (APS) agencies nationwide is self-neglect (SN). Researchers collaborated with Oklahoma APS to understand characteristics of community-dwelling clients reported to APS for alleged SN, and examine predictors associated with whether cases were validated by APS case managers. Baseline phone interviews were conducted with 188 clients reported to APS for self-neglect during the COVID-19 pandemic. A logistic regression was run to examine the effects of age, gender, income, race, education, cognitive status, ADL impairment, depression, recent nursing home placements, hospitalizations and falls on the likelihood that participants’ allegation of SN was validated. The model was statistically significant, χ2(12) = 26.67, p = .009. It explained 24.1% of the variance in case validation and correctly classified 81.8% of cases. Having a history of being in a short-term nursing home and having a history of falls was significantly associated with the increased likelihood of having a validated SN allegation. However, a history of hospitalization was significantly associated with a reduction in the likelihood of having a validated SN allegation. These preliminary findings suggest that perhaps hospital discharge planners may be assisting SN patients to access home-and-community based services (HCBS) early on to address their SN so it is not validated by APS compared to nursing home discharge planners. Implications for practice include educating APS staff on predictors of SN and collaborating with community-based partners like short-term rehabilitation facilities to address SN issues to reduce the likelihood of having a case validated by APS.

